# Integrated care pathway for the management of diabetic foot disease: a position statement from the Italian Diabetic Foot Study Group

**DOI:** 10.1007/s00592-026-02741-1

**Published:** 2026-07-13

**Authors:** Laura Giurato, Marco Meloni, Alessia Scatena, Roberto De Giglio, Giuseppe Murdolo, Marco Mastinu, Maurizio Gicchino, Desiree Gabriele, Roberto Anichini, Cesare Miranda, Luigi Uccioli, SID-AMD Diabetic Foot Study Group

**Affiliations:** 1 Division of Endocrinology and Diabetes, Department of Surgical Sciences, CTO Hospital, University of Rome Tor Vergata, Rome, Italy; 2 Department of Systems Medicine, University of Rome “Tor Vergata”, Rome, 00133 Italy; 3 Division of Endocrinology and Diabetology, Department of Medical Sciences, Fondazione Policlinico “Tor Vergata”, Rome, Italy; 4 Diabetology Unit, Health Authorities South East Tuscany, San Donato Hospital, Arezzo, Italy; 5 Diabetic Foot Functional Unit, Paderno Dugnano Milan, San Carlo, Italy; 6 Department of Endocrinology and Metabolism, Santa Maria Della Misericordia Hospital, Perugia, Italy; 7 Unit of Metabolic Diseases and Diabetology, San Martino Hospital, Oristano, Italy; 8 Unit of Endocrinology and Metabolic Diseases, University Hospital L. Vanvitelli, Naples, Italy; 9 Studio Podologico Gabriele, Sora, Italy; 10 Diabetology Unit, Center of Excellence for Diabetic Foot Care, Tuscany Central Local Health Authority, Pistoia, Italy; 11 Clinic of Endocrinology and Metabolism Diseases, ASFO, Pordenone, Italy

**Keywords:** Diabetic foot, Care pathways, Diabetology setting, Multidisciplinary care

## Abstract

Diabetic foot is one of the most serious chronic complications of diabetes mellitus and reflects the systemic burden of the disease. Preventive strategies and structured management pathways are therefore essential elements of diabetes care. Lifetime occurrence of foot ulceration has been reported in nearly one-third of individuals with diabetes, and these lesions account for most non-traumatic lower-extremity amputations. Furthermore, the estimated five-year mortality following ulceration is 30%. This document aims to ensure an appropriate, multidisciplinary care pathway for patients with diabetic foot, tailored to clinical complexity and aligned with the different levels of care of Italian diabetes centers. The integrated model of diabetic foot care is based on the level of care that diabetes centers (local and hospital) can provide, including available personnel, expertise, and diagnostic and therapeutic resources to manage varying degrees of clinical complexity. Accordingly, three levels of care complexity, low, medium, and high, have been defined, with corresponding key diagnostic and therapeutic activities and healthcare professionals involved.

## Introduction

 Diabetic foot disease is among the most challenging complications of diabetes, significantly affecting patients’ quality of life, survival, and the use of healthcare resources. Over a lifetime, up to one-third of individuals with diabetes will develop foot complications, which contribute substantially to hospital admissions related to diabetes. Globally, foot ulceration is estimated to affect approximately 18 million people [1–3].

Ulceration represents a critical stage in the progression of diabetic foot, frequently preceding lower-limb amputation [4]. Despite advances in diagnostics and treatment, prognosis remains poor: the five-year survival rate is around 30% in patients with active diabetic foot ulcers, and following a major lower-limb amputation, mortality may reach 70% [5]. Complete healing occurs within 12 weeks in approximately one-third of ulcers [6]. While recurrence is common, affecting about 42% of patients within the first year and nearly 65% within three years [7, 8].

The development of foot ulcers is a result of multiple interacting factors. Peripheral neuropathy, structural foot deformities, and peripheral arterial disease are the primary causes of ulceration. Chronic kidney disease, especially dialysis, further raises the risk of ulcers [9, 10].

Optimal outcomes require early identification of at-risk patients, proactive preventive strategies, and a structured, multidisciplinary approach integrated into a coordinated care pathway [11].

Within Italy, the National Diabetes Plan (PND, 2012) identifies the diabetic foot as a key priority for improving diabetes care. The plan advocates for systematic foot risk assessment, timely intervention, enhancement of professional skills in foot care, and the development of coordinated care models to enable quick recognition and management of complications. Prevention of amputation involves regular foot and footwear assessments, early management of existing lesions, multidisciplinary treatment strategies, prompt detection and intervention for peripheral arterial disease, including revascularization when necessary, and ongoing follow-up for individuals with a history of ulceration [12].

## Methods

The Italian diabetology network currently encompasses various types of facilities, including community outpatient services, hospital-based diabetes clinics, and diabetic foot reference centers.

Management of diabetic foot disease shows considerable variation across regions and healthcare settings, reflecting differences in socioeconomic context, local resources, and care standards. National surveys have identified persistent challenges, such as delayed referral to specialized foot units and limited access to urgent surgical procedures [13]. 

To evaluate current practice, we analyzed data from the Annals of the Associazione Medici Diabetologi (AMD), a nationwide quality assessment program active since 2006. In 2024, the records indicated that structured foot evaluation was documented in only 15.8% of patients [14].

To fill these gaps, a structured diagnostic and therapeutic pathway for the care of the diabetic foot should be integrated into the routine management of diabetes. Accurate and timely staging is essential to assign patients to the most appropriate level of care, ranging from routine foot risk assessment and early detection of ulcers to more complex interventions and specialist management.

### Objective of the document

The current document aims to offer practical guidance for diabetologists in the management of diabetic foot disease. The focus is on three key aspects: early identification of patient-specific risk factors to prevent ulcer formation (primary prevention), coordinated multidisciplinary management of active lesions according to their severity (acute phase), and structured long-term follow-up for patients with prior ulcers or amputations (secondary prevention).

Implementation of systematic foot screening across all services within the national diabetes care network is essential. Effective management relies on close coordination among centers with different levels of expertise, with the intensity of interventions tailored to each center’s capacity to manage different clinical scenarios, including the presence and severity of ulcers, ischemia, and infection. (see Table 1)

**Table 1:** Integrated management of diabetic foot according to care complexity

Complexity level	Predominant clinical condition	Main intervention	Place of care	Care objective
Low complexity	No ulcer; loss of protective sensation; absent or mild peripheral artery disease	Yearly screening, primary and secondary prevention; patient education	Community outpatient clinic, primary care diabetologist	Prevention of foot ulcer and early recognition of risk factors. Secondary prevention
Medium complexity	No deep ulcers, mild infection; recurrent ulcers; moderate peripheral artery disease (not requiring lower limb revascularization)	Wound care, advanced dressings, vascular assessment, and close follow-up	Hospital-based diabetes centers or diabetic foot clinics	Primary prevention: wound healing and prevention of ulcer recurrence
High complexity	Deep ulcers, severe infections, critical limb-threatening ischemia, and Charcot foot	Surgical treatment, Revascularization, multidisciplinary team, hospitalization	Referral center with Diabetic Foot Unit	Limb preservation, High level of wound management, including advanced surgical aspects, revascularization, integrated management of diabetic complications, and prevention of ulcer recurrence

The table shows the management of diabetic foot according to the degree of clinical complexity

## Low-complexity diabetology settings: integrated management of the diabetic foot

### Essential criteria

Diabetology services operating in outpatient settings must ensure early diagnosis of diabetic foot disease through screening for risk factors of foot ulcer, while also delivering primary and secondary prevention interventions.

### Essential care requirements

A core team consisting of a diabetologist and a trained, dedicated nurse and/or podologist (where available).

- Direct access, through established referral pathways, to medium- and high-complexity centers in the event of complications such as ulceration, infection, or ischemia.
- A dedicated outpatient facility for patient screening and follow-up visits.
- Diagnostic tools for basic screening: 10-g monofilament, 128-Hz tuning fork or biothesiometer, reflex hammer for Achilles reflex assessment, and continuous-wave (CW) Doppler with visual waveform for ankle–brachial index (ABI) measurement.

### Health care professionals

**Diabetologist**: Responsible for regularly assessing risk assessment to determine the risk category and define the frequency of individual follow-up. The diabetologist also prescribes and/or recommends appropriate footwear and insoles according to ulcer risk category and clinical history (primary and secondary prevention).

**Dedicated nurse**: Responsible for performing patient screening, managing periodic monitoring, and contributing to patient education.

**Trained podologist**: Performs patient screening and education, manages pre-ulcerative lesions.

### Time allocation

When a dedicated outpatient setting is not available, screening activities may be organized within predefined appointment slots. This approach avoids prolonging routine diabetes visits and promotes structured follow-up.

### Ulcer risk stratification

Patients with diabetes are at varying risk for foot ulceration, and the International Working Group on the Diabetic Foot (IWGDF) suggests stratifying this risk based on loss of protective sensation (LOPS), the presence of peripheral arterial disease, foot deformities, previous ulceration or amputation, and advanced renal disease treated with dialysis [15]. 

Risk stratification guides the definition of appropriate follow-up intervals and enables regular surveillance aimed at the timely identification and management of associated risk factors.

### Foot inspection in patients with diabetes

Key preventive measures, integral to the routine diabetes consultation, include:

- Assessment of toe deformities (claw toes, hammer toes, hallux valgus), flatfoot or high-arched (cavus) foot, and prominent metatarsal heads.
- Evaluation of active ulcers or pre-ulcerative signs, including hyperkeratosis, hematomas, subcutaneous hemorrhages, and skin fissures.
- Assessment of footwear, including size, shape, structural deformities, and sole wear.
- In the presence of a foot ulcer, the patient should receive clear instructions on local wound care (appropriate dressing and effective offloading) and be referred to a specialized diabetic foot center according to clinical severity.
- For a superficial ulcer limited to the epidermis and dermis, without necrosis, ischemia (with palpable peripheral pulses), or clinical signs of infection, local treatment may be started, with referral to an intermediate-complexity center within two weeks.
- If the ulcer is associated with necrosis, absent peripheral pulses suggestive of ischemia, or clinical signs of infection, prompt referral to a medium- to high-complexity center is required, ideally within 48–72 h.

### Patient communication

At the end of each visit, patients should receive structured educational materials, involving caregivers when necessary, to support foot care education and prevent foot ulceration. Education should include information on:

- Daily foot inspection.
- Warning signs that should be promptly reported to the physician/diabetologist.
- Protective behaviors, such as the use of appropriate footwear and avoidance of barefoot walking.
- Regular podiatric care.

### Absence of an ideal setting

When dedicated facilities or trained nursing staff are not available, a diabetologist should at least perform an initial foot assessment during the routine diabetes consultation. This assessment should include:

- Physical examination of the foot to exclude active lesions and identify potential pre-ulcerative conditions, such as hyperkeratosis and foot deformities;
- Palpation of peripheral pulses, including the dorsalis pedis and posterior tibial arteries;
- The Ipswich Touch Test;
- Evaluation of co-morbidities, specifically ESRD (Table 2).

**Table 2:** Management of diabetic foot in low-complexity diabetology settings

Area of intervention	Specific activities
Prevention and screening	- Yearly ulceration risk assessment for all diabetic patients - Physical examination of the foot, including skin and nail inspection - Palpation of peripheral pulses (dorsalis pedis and posterior tibial); performance of ABI - Sensory testing with monofilament, tuning fork, and reflexes - Risk classification according to IWGDF - Prescription of therapeutic footwear and insoles
Internal medicine assessment	-Optimization of metabolic control - Management of cardiovascular and renal risk factors - Antiplatelet/anticoagulant therapy - Statin therapy
Therapeutic education and self-care	-Education of patient and caregiver on hygiene, nail care, hydration, and daily foot care -Instructions on proper use of footwear and insoles -Information on warning signs (calluses, blisters, color changes, pain)
Management of pre-lesions	- Treatment of calluses and pre-ulcerative lesions (podologist) - Regular podiatric care
Follow-up and periodic monitoring	- Organizing follow-up care based on risk level (every 1–3, 6, or 12 months) - Periodic reassessment of patient education - Monitoring the effectiveness of preventive measures and educational interventions
Interdisciplinary collaboration and continuity of care	- Structured communication with the general practitioner (GP) - Prompt referral to medium–high complexity centers in case of lesions, infection, or suspected ischemia - Participation in training programs

The table illustrates the intervention areas and specific activities for diabetic foot management in a low-complexity diabetology center

## Medium-complexity diabetology settings: integrated management of the diabetic foot

### Structural and organizational requirements

These outpatient facilities must be equipped with dedicated expertise in diabetic foot management, encompassing early diagnosis, including ulcer risk stratification, as well as the management of uncomplicated lesions.

### Essential care assumptions

- Availability of organized and dedicated clinical areas;
- Availability of trained, experienced, and dedicated professionals;
- Availability of radiological and cardiological services;
- Direct pathways to high-complexity centers for advanced care.

### Health care professionals

**Diabetologist**: team leader for clinical management within the multidisciplinary foot team and plays a central role in driving diagnostic and therapeutic decision-making.

**Dedicated nurse with wound care expertise**: ulcer prevention and co-management of active wounds.

**Trained podologist**: risk stratification, patient education, and management of pre-ulcerative lesions.

**Cooperating specialists**: Nephrologist, Cardiologist, Vascular Surgeon, Orthopedic Surgeon, Infectious Disease Specialist, Radiologist, including an interventional radiologist.

### Management of patients without active lesions

In a medium-complexity center, the diabetologist plays a key role in the primary and secondary prevention (including monitoring of ulcer recurrence). Mandatory steps include:

- Ulcer prevention and risk stratification;
- Therapeutic education;
- Prescription of therapeutic footwear and insoles according to the individual risk of ulcer development and clinical history;
- Regular follow-up.

### Management of patients with active lesions

The diabetologist, in cooperation with wound care nurses and trained podologists, is the main professional responsible for the integrated management of foot lesions, supported by specialist consultants in a multidisciplinary care pathway.

Defining an ulcer accurately is crucial, taking into account the depth of tissue involvement, the presence of ischemia, and the risk of infection. Frequently applied classification methods include the Wound, Ischemia, and foot Infection (WIfI) system and the IWGDF/IDSA criteria [16, 17]. 

At the end of each treatment, the diabetologist ensures that patients or caregivers receive specific education aimed at preventing ulcer recurrence, verifies adherence to protective measures, such as the regular use of therapeutic footwear, and schedules appropriate follow-up (see Table 3).

### Priority access to high-complexity centers

Patients with complex clinical conditions, such as ischemic lesions requiring surgical or endovascular revascularization or infected lesions requiring surgical interventions (e.g., abscess drainage, debridement, ulcerectomy, sequestrectomy, or minor amputations), should have priority access to high-complexity service, as these procedures cannot be performed in medium-complexity care settings.

Additionally, if no improvement is observed after 4–6 weeks of standard care (defined as the absence of reparative signs and less than 50% reduction in ulcer diameter), the patient should be referred to a high-complexity diabetic foot center.

**Table 3:** Management of diabetic foot in medium-complexity diabetology settings

Area of intervention	Specific activities
Internal medicine assessment	- Optimization of metabolic control - Management of cardiovascular and renal risk factors - Antiplatelet/anticoagulant therapy -Statin therapy
Care of patients without foot lesions	- Periodic monitoring of patients with neuropathy and/or peripheral artery disease - Treatment of hyperkeratosis and other pre-lesions - Prescription of therapeutic footwear and insoles - Cooperation with a podologist for regular care
Care of the foot with an ulcer	-Initial assessment of lesion (size, depth, infection, ischaemia) -Infection: evaluation of local and systemic signs, exudate, fever, etc. (IWGDF/IDSA classification) - Wound classification/score (WIfI) - Debridement and dressing with advanced devices (including reconstructive surgery) - Prescription of offloading devices (removable or custom-made)
Instrumental and clinical diagnostics	-Peripheral artery disease assessment: pulses; ABI -Advanced neuropathic assessment (vibration with biothesiometer; thermal sensitivity)
Management of mild–moderate infection	- Identification of local inflammatory signs - Microbiological evaluation (swabs or biopsy) - Selection of appropriate dressing
Management of severe infection	- Initial surgical intervention for abscess or fistula drainage. - Activation for urgent admission to a high-complexity center
Local therapy	-Debridement of ulcerative lesions -Selection of appropriate dressing -Selection of the correct offloading device
Healing support	- Education of patient and caregiver on the use of acute phase orthoses and lesion management -Prescription of therapeutic footwear and insoles -Scheduling of follow-up (diabetologist and podologist)
Secondary prevention	- Education of the patient on foot care - Use of therapeutic footwear with insoles - Regular follow-up with podologist - Motor rehabilitation

The table illustrates the intervention areas and specific activities for diabetic foot management in a medium-complexity diabetology center

## High-complexity hospital diabetology settings (foot unit)

### Multidisciplinary team approach

These centers, structured for the management of complex diabetic foot cases, are required when severe foot ulcers, including those with vascular impairment and/or deep infections, require integrated expertise and access to advanced technologies.

Reference centers for the management of complex diabetic foot cases should provide multidisciplinary care, with integrated skills for managing severe and complex wounds, vascular complications, and deep infections, through access to advanced diagnostic and therapeutic technologies.

### General care requirement

- Dedicated inpatient beds, with elective admission and emergency department access.
- Direct access for patients referring from lower-complexity care levels or the community.
- Management of complex wounds, including moderate-to-severe infection, osteomyelitis, with access to a dedicated surgical environment/operating room.
- Admission for revascularization procedures in patients with critical limb-threatening ischemia.
- Dedicated access to ordinary radiological imaging, including foot X-ray, magnetic resonance imaging (MRI), duplex color ultrasound, contrast tomography (CT) angiography, and skin perfusion (oximetry).
- Access to advanced therapies, such as negative-pressure therapy (NPT), tissue engineering (dermal-epidermal substitutes), and regenerative therapies (autologous cell therapy).
- Protocols for prolonged intravenous therapy in a Day Hospital setting (e.g., antibiotics).
- Management of Charcot foot.

### Multidisciplinary team

**Diabetologist**: management of the clinical-care pathway, ensuring optimal metabolic control and integration of specialists according to clinical needs. A diabetologist with surgical expertise may be indicated to perform demolitive and/or reconstructive procedures below the ankle when other surgical specialists (vascular, general, or orthopedic) are not available.

**Vascular surgeon / interventional radiologist**: responsible for revascularization procedures in the management of critical limb ischemia.

**Infectious disease specialist / microbiologist**: management of antimicrobial therapy.

**Radiologist**: management of advanced imaging studies.

**Foot surgeon / orthopedic surgeon**: intervenes in the treatment of complex surgical cases, including reconstruction of severe deformities, management of deep infections, and amputations.

**Plastic surgeon**: intervenes in reconstructive procedures as needed.

**Trained podologist**: provides foot management in collaboration with diabetologists, including ulcer management, patient screening, follow-up, podiatric care, orthotic interventions, and patient education.

**Orthotic technician**: responsible for managing offloading devices during the acute phase and for therapeutic footwear and insoles after wound healing.

**Physiatrist and physiotherapist**: ensure functional recovery following surgical interventions or wound healing.

**Expert nurse (case manager)**: supports therapeutic education and wound care in collaboration with diabetologists, including patient screening, follow-up, wound management, and the application of specific wound dressings.

High-complexity centers receive patients from lower-complexity services and discharge patients once the complex phase of care has been completed.

**Priority pathway for critical limb ischemia (CLTI)**: Critical limb ischemia should follow a dedicated care pathway, including the evaluation of the patient’s cardiovascular and renal status. The diabetologist, acting as case manager, coordinates the whole patient strategy of care in association with the vascular surgeon, interventional cardiologist, and interventional radiologist, who are directly responsible for their respective procedures [18]. 

### Management of complex infections

Integrated and timely management of soft tissue and bone infections is essential to reduce the risk of amputation. The diabetologist collaborates with the infectious disease specialist to manage targeted antibiotic therapy, with the vascular specialist or interventional radiologist to select the appropriate revascularization procedure, and with the radiologist to coordinate diagnostic investigations. A diabetologist with surgical expertise may independently perform wound debridement and surgical interventions when indicated.

**Regenerative medicine in diabetic foot pathology**: High-complexity centers have access to advanced therapeutic options, including regenerative therapies such as growth factors, stem cell and cellular therapies, as well as dermal and bone substitutes, biomaterials, and bioactive scaffolds for tissue regeneration [19, 20] (Table 4). 

**Table 4:** Management of diabetic foot in high-complexity centers

Area of intervention	Specific activities
Internal medicine assessment	- Optimization of metabolic control - Management of cardiovascular and renal risk factors - Antiplatelet/anticoagulant therapy -Statin therapy - Renal and cardiovascular protective/preventive therapy
Advanced diagnostics	- Vascular assessment with dedicated imaging (color Doppler ultrasound, CT angiography); Oximetry; Diagnosis of osteomyelitis and Charcot foot (MRI, scintigraphy) - Microbiological diagnostics - Biomechanical and baropodometric analysis
Revascularization	-Multi-level percutaneous angioplasties -Peripheral bypasses and hybrid techniques -Monitoring of tissue perfusion post-procedure
Early re-evaluation after Revascularization	- Assessment of procedure outcomes: evaluation of final angiograms, oximetry, and arterial duplex ultrasound -Intensive control of cardiovascular/metabolic risk factors
Management of moderate–severe infections	- Microbiological diagnosis - Empiric and isolated targeted antibiotic therapy - Osteomyelitis management
Regenerative therapies	- Application of dermal substitutes - Growth factors, biomaterials, and stem cells - Use of bioactive scaffolds for tissue regeneration
Wound management	- Advanced dressings - Negative-pressure therapy - Offloading orthoses
Foot surgery and limb-salvage procedures	- Surgical debridement - Bone stabilization - Deformities correction - Flap surgery and integrated reconstructive plastic surgery
Prevention and rehabilitation	- Therapeutic footwear and insoles - Functional rehabilitation programs
Multidisciplinary follow-up |	- Integrated clinical monitoring, metabolic and vascular control, ulcer recurrence prevention - Continuity of care with local healthcare services

The table illustrates the intervention areas and specific activities for diabetic foot management in a high-complexity diabetology center

The diabetologist should ensure specific expertise in the clinical management of complex diabetic foot. To effectively coordinate the multidisciplinary team, the diabetologist should know different clinical fields (vascular surgery, wound care, antibiotic therapy, etc.), necessary to achieve shared therapeutic decisions and ensure the appropriate care pathway for each patient [21].

Furthermore, the diabetologist should remain up-to-date on the various therapeutic options and take responsibility for the education and training of any members included in the multidisciplinary team.

## Orthotic therapy in patients with diabetes

### Indications for shoes and insoles in primary prevention

In patients with diabetes, the use of inappropriate footwear is a major contributor to foot ulceration. While unsuitable shoes may trigger ulcer development, appropriate footwear can prevent it. Therapeutic footwear and custom-made insoles must meet functional and protective standards to ensure plantar load redistribution and protection of high-risk areas.

Therapeutic shoes and custom insoles should satisfy specific functional and protective requirements to ensure even distribution of plantar load, prevent trauma by accommodating foot deformities, and promote safe ambulation [22–24]. 

Footwear is an essential therapeutic device in the management of the diabetic foot, as important as glycemic control and ulcer care. As a true therapeutic intervention, it must be tailored to the individual patient and their clinical profile. The selection of shoes and insoles should be guided by ulcer risk level, according to the International Working Group on the Diabetic Foot (IWGDF) classification, while also considering physical activity, lifestyle, and individual patient needs [3]. 

#### Risk 0 – very low

*Characteristic*: absence of neuropathy, peripheral arterial disease, and deformities.

- Normal, comfortable, well-fitting shoes (not tight-fitting).
- Recommended: patient education on foot self-care.

#### Risk 1 – low

*Characteristic*: foot with neuropathy or peripheral arterial disease without deformity.

- Protective footwear.
- No protruding seams.
- Padded or soft edges.
- Lace or Velcro closure over the dorsum.
- Flexible sole.
- Insole: single-layer, pressure-dissipating.
- Recommended: patient education on foot self-care.

In this condition, where loss of protective sensation or arterial circulation impairment may render the patient more vulnerable to trauma, appropriate footwear increases foot protection and reduces the risk of ulceration. In clinical practice, shoes with the above characteristics may be recommended.

#### Risk 2 – moderate

*Characteristic*: foot with deformities of varying degree, with neuropathy or peripheral arterial disease.

- Preventive footwear: low or high.
- Semi-rigid sole.
- Upper: thermoformable or self-molding, with no internal seams.
- Toe box: compatible with the morphology of the forefoot and toes.
- Extra-depth to accommodate insoles and deformities, particularly in the forefoot.
- Custom multi-layer insole molded from total-contact cast.

For this risk class, where deformities are associated with sensory loss and peripheral arterial disease, shoes and insoles should be prescribed according to the above specifications.

### Indications for shoes and insoles in secondary prevention

#### Risk 3 – high

*Characteristic*: foot with deformities of varying degree, peripheral neuropathy or peripheral arterial disease, history of previous ulceration and/or amputation.

- Low or high shoes with rigid/semi-rigid counters (prefabricated or custom).
- Rigid sole.
- Upper: thermoformable or self-molding with no internal seams.
- Toe box consistent with the morphology of the forefoot and toes.
- Extra-depth to accommodate insole and deformities, particularly in the forefoot.
- Custom multi-layer, total-contact, enveloping insole.
- Possible insole filler at amputation sites (e.g., first ray).

For this risk class, which defines secondary prevention, it is essential to prescribe footwear and insoles with the specified features to prevent recurrent ulceration. Patients on dialysis also fall into this category, as dialysis is an independent risk factor for ulceration. Footwear and insoles should therefore be prescribed based on the presence or absence of deformities.

For at-risk patients, therapeutic footwear must be worn in all settings, both outdoors and at home, to ensure continuous foot protection. Home-use models are available, designed to accommodate insoles and featuring a closed heel to provide protection and pressure offloading.

## Recommended digital tools

Diabetologists are advised to use an electronic medical record system (such as MetaClinic) to document and characterize patients during both preventive and acute phases. This structured, standardized, and integrable digital tool supports primary and secondary prevention as well as acute diabetic foot management, improving clinical effectiveness, traceability, and quality of care.

## Training of healthcare professionals according to level of care

Healthcare professionals involved in diabetic foot management should receive training tailored to their specific role and the stage of disease.

The diabetologist must be trained in the assessment and management of the diabetic foot according to disease progression and complexity, through university-level Master’s programs and specialized courses, with periodic refresher training. Additionally, at medium- and high-complexity care levels, diabetologists should acquire basic and emergency surgical skills through dedicated specialized training programs.

Nurses and podiatrists should receive specialized training in diabetic foot management through university-level Master’s programs and targeted courses. Scientific societies play a key role in professional education and training.

Diabetologists involved in diabetic foot care, in addition to their clinical expertise, should actively contribute to the training of other healthcare professionals involved in patient management.

**Table 5:** Diabetologist’s role – training dimension

Training area	Objective	Role of the diabetologist	Practical modality
General practitioners (GPs)	Early screening and timely referral	Train on screening and referral according to risk	ECM sessions
Community nurses	Surveillance and territorial prevention	Train in monofilament use, ABI, patient education, and dressing according to lesion status	ECM/FAD courses, practical simulations
Emergency staff and other specialists	Emergency management of diabetic foot	Develop emergency protocols, workshops on severe infection and ischemia	ECM, on-site sessions, shared protocols

The table shows the role of the diabetologist in the training pathway of other healthcare professionals involved in diabetic foot management

## Discussion

The organization of diabetic foot care should be structured according to the level of clinical complexity, with clearly differentiated responsibilities across care settings.

At the low-complexity center, the primary objective is risk containment. This requires systematic foot risk stratification integrated into routine diabetes visits, alongside structured patient education focused on daily inspection, hygiene, and early recognition of warning signs. In this setting, prevention is not an adjunct activity but the principal clinical mandate, aimed at reducing both first-time ulceration and recurrence.

As clinical demands increase, medium-complexity services must expand their scope beyond prevention to include the management of non-complicated acute lesions. Here, the diabetologist assumes direct responsibility for early therapeutic interventions while maintaining vigilance for signs that require escalation of care. The ability to distinguish cases suitable for local management from those requiring referral becomes a critical competency.

High-complexity or specialized centers, equipped with dedicated Diabetic Foot Units, provide the structural and professional framework necessary for multidisciplinary management of advanced disease. In this context, the diabetologist functions as clinical coordinator, ensuring diagnostic coherence, therapeutic prioritization, and continuity across different specialist contributions. Leadership in this setting does not imply exclusivity of action but responsibility for integrating expertise within a multidisciplinary care plan [25]. 

Delays in accessing specialist care are largely due to organizational issues, including a lack of standardized referral criteria, a lack of community-based risk stratification, failure to recognize the lesion early, and the absence of clearly structured care pathways [26]. 

Across all levels of care, the diabetologist maintains a pivotal role; however, the intensity and nature of clinical responsibility should adapt to the patient’s degree of complexity. This dynamic allocation of responsibilities prevents both under-treatment in high-risk patients and unnecessary centralization of low-risk cases.

This document aims to outline a model of diabetic foot care as an example of a patient management pathway based on clinical risk stratification, in terms of lesion prevention and its timely management according to the level of clinical care, resources, timely referral, and proportional intensity of intervention.

## Conclusion

Stratifying patients based on clinical complexity allows for more efficient distribution of care between community services and hospitals.

Each level of care plays a pivotal role in the management of diabetic foot disease. Preventive strategies, ranging from individual risk stratification for every person with diabetes to the treatment of pre-ulcerative conditions and the prescription of appropriate footwear, represent the most effective measures to reduce the risk of ulcer development.

Early identification of ulceration and its prompt management contribute to lowering the associated morbidity and mortality.

Patients with high clinical complexity benefit from management in specialized multidisciplinary centers, where access to advanced therapies can be guaranteed.

Finally, implementing standardized care pathways and structured training for healthcare professionals supports continuity of care and improves clinical efficacy at all levels of diabetes care (see Table 6).

**Table 6:**
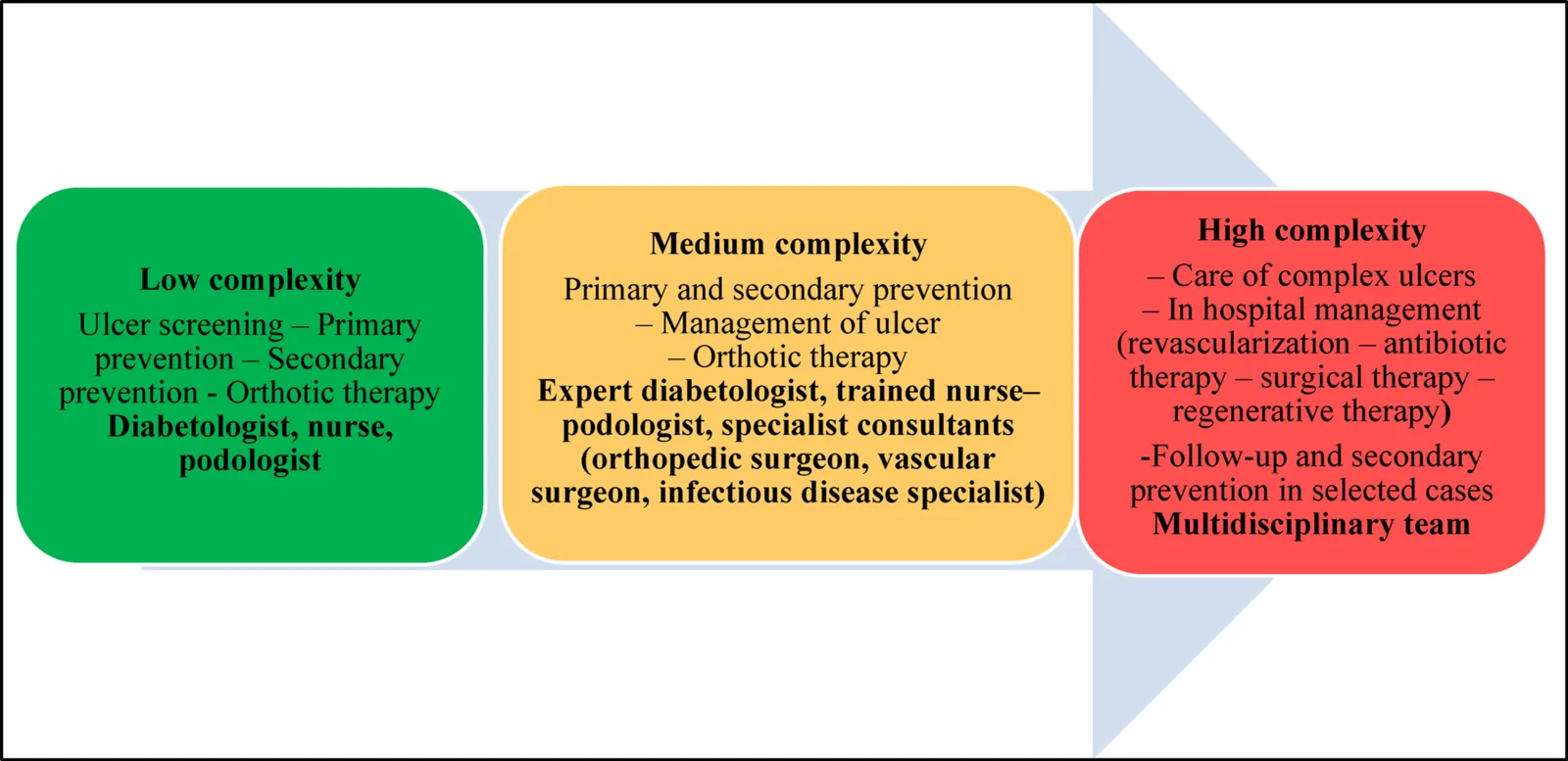
Diabetic foot care pathway

This table shows the management of diabetic foot based on the level of care intensity of diabetes centers
